# Editorial: Advances in systems neurogenetics

**DOI:** 10.3389/fnins.2025.1752179

**Published:** 2026-01-06

**Authors:** Paul Carney, Bing Zhang

**Affiliations:** 1 Departments of Pediatrics and Neurology, University of Missouri, Columbia, MO, United States; 2 Division of Biological Sciences, University of Missouri, Columbia, MO, United States

**Keywords:** gene, neuroscience, development, *Drosophila*, systems

Systems neurogenetics seeks to explain how genetic variation shapes neural function, vulnerability, and clinical phenotype across the lifespan. By integrating molecular pathways, cellular networks, circuit physiology, and organismal behavior, this field provides a framework for understanding neurological disorders that exhibit complex interactions between genes, environment, and developmental timing. The articles included in this Research Topic represent four complementary domains: environmental toxicology, neuronal gene-regulatory timing, neurocutaneous syndromes with heterogeneous clinical expressivity, and transcriptomic dissection of monogenic leukodystrophy. Together, these contributions highlight the mechanistic depth and clinical relevance that arise when neurogenetic questions are approached from a systems perspective.

De Donno et al. examine chronic cadmium exposure using *Drosophila melanogaster* to model how environmental toxins interact with neuronal aging. Cadmium is a well-recognized heavy metal contaminant with established epidemiological associations with Alzheimer's disease, Parkinson's disease, and amyotrophic lateral sclerosis. In this study, cadmium exposure reduces lifespan, worsens learning and memory, and produces neurodegenerative features in flies. Mechanistically, cadmium disrupts ribonucleoprotein granules by altering liquid–liquid phase separation, a biophysical mechanism increasingly understood as central to neurodegenerative diseases. Aberrant phase transitions of RNA-binding proteins are implicated in ALS, FTD, and tauopathies. By demonstrating that cadmium perturbs this process *in vivo*, the authors position environmental neurotoxicity within a unifying mechanistic pathway that links metabolic stress, impaired RNA handling, and age-related cognitive decline. This work underscores the power of *Drosophila* as a system that allows aging, synaptic function, and molecular organization to be interrogated across scales.

The study by Yin et al. explores the interaction between circadian regulatory genes and long-term memory formation. Using precise, inducible genetic tools in *Drosophila*, the authors demonstrate that expression of clock genes in the dorsal anterior lateral neurons is essential for memory consolidation but not retrieval. These neurons are required for olfactory memory but are not part of the classical central circadian clock. Acute suppression of *clock* or *cycle* genes after training impairs three-day memory, whereas induction prior to testing does not, supporting a temporally restricted requirement during consolidation. Intriguingly, global mutants for the same genes exhibit normal memory, suggesting that systems-level compensation across the circadian network masks the deficit when disruption occurs developmentally. This distinction highlights a fundamental concept in systems neurogenetics: cell-type-specific perturbations can reveal roles obscured in whole-organism mutants by circuit-wide buffering. The study integrates molecular rhythmicity, synaptic plasticity, and circuit function, offering mechanistic clues relevant to sleep-dependent memory processes observed across species.

Ren et al. focus on neurofibromatosis type 1, a complex Ras-MAPK pathway disorder characterized by extreme phenotypic variability even among individuals sharing identical NF1 variants. The case they describe illustrates this challenge clearly. A child with epilepsy harbors two NF1 variants: a novel frameshift mutation inherited from her mother and a splice-site change inherited from her father. Despite carrying the same frameshift mutation, the mother developed tumors without epilepsy, while the father, who carries the splice-site variant, remains unaffected. Through genetic testing and structural modeling, the authors conclude that the frameshift mutation is likely pathogenic, whereas the splice-site variant appears benign. This analysis demonstrates how clinical interpretation requires integrating molecular prediction, family segregation, protein modeling, and phenotype correlation. Importantly, the phenotypic discordance between mother and daughter reinforces the concept that NF1 severity is shaped by context-dependent factors, including modifier genes, developmental windows, and tissue-specific sensitivity to Ras-MAPK dysregulation. This case contributes to the broader understanding that genotype alone cannot reliably forecast neurologic outcomes in NF1.

Fu et al. address the problem of variable expressivity in X-linked adrenoleukodystrophy by studying a family with an exon-2 deletion in *ABCD1*, including monozygotic twins discordant for childhood-onset disease. Using whole-blood transcriptome sequencing, the authors identify differentially expressed genes and enriched pathways associated with onset timing and disease severity. Candidate genes include *C4BPA, TPBG, CHST15*, and *SMAD1*, with enrichment for pathways involving calcium homeostasis, immune signaling, and membrane regulation. By incorporating data from asymptomatic carriers and an unaffected homozygous relative, the study maps transcriptional patterns that may serve as modifiers or early indicators of clinical trajectory. Although peripheral blood cannot fully reflect central nervous system pathology, the systems-level approach provides valuable hypotheses regarding resilience and vulnerability in monogenic leukodystrophy. The work exemplifies how multi-individual transcriptomic comparison can illuminate regulatory differences that shape disease onset, even among genetically identical individuals.

Across these contributions, several unifying themes emerge. First, multiscale integration is essential for understanding neurogenetic disorders. Whether examining LLPS disruption in toxic aging, circadian gene cycling in memory circuits, Ras-MAPK dynamics in NF1, or transcriptional modifiers in ALD, each study demonstrates that pathogenic mechanisms operate across molecular, cellular, and systems levels. Second, genetic background and context critically modify clinical expression. The NF1 family and ALD twins illustrate how identical variants can lead to divergent outcomes through modifier networks and developmental timing. Third, model organisms provide irreplaceable mechanistic insight. The *Drosophila* studies highlight how conserved genetic pathways can be interrogated with temporal and spatial precision, revealing principles relevant to human disease. Fourth, integrating clinical data with molecular and computational tools strengthens diagnostic interpretation and may guide future therapeutic strategies.

Together, these articles reflect the expanding capacity of systems neurogenetics to dissect complex neurological disorders. As technologies such as single-cell sequencing, spatial transcriptomics, high-resolution circuit mapping, and machine-learning-based phenotyping continue to advance, they will further enhance our ability to connect genetic variation with neural function and clinical outcome. This Research Topic demonstrates that mechanistic understanding emerges most powerfully when genetic, molecular, and systems-level analyses are combined, reinforcing the promise of precision approaches in neurology and neurodevelopmental medicine.

